# Modern Sociology

**Published:** 1902-05-24

**Authors:** 


### Modern Sociology.

### THE KING'S DINNER.

The kindly wish of the King to give a dinner in commemoration of his Coronation to every poor person resident in the metropolis of the Empire on July 5th will be appreciated by all classes. No one can doubt that if it is possible to secure that every poor person shall enjoy a festival of this kind at the Coronation every generous-minded subject will rejoice. From inquiries we have made there is, however, reason to fear that, unless those responsible for the arrangements become more fully alive to the difficulties to be encountered in so vast an undertaking, in some districts at any rate, instead of a King's feast there may be a municipal failure. The arrangements have been left by the King to the municipal authorities, acting under the direction of a committee sitting at the Mansion House. The practical difficulties to be overcome before universal success can be obtained are serious indeed. Let us examine them in detail.

At the first blush, having regard to the facilities afforded by the cheaper restaurants and the prices charged, an allowance of 1s. 2d. as the cost of each dinner would appear to be ample. In fact, owing to the refusal in certain districts of the larger contracters to undertake the catering, and to the absence of the necessary appliances for dining many hundreds of people in a given place, something like a deadlock in the arrangements is threatened. To provide a good dinner every day in the week at a restaurant is a relatively simple matter. But to arrange to give one dinner on one occasion to hundreds of people in many places where no such facilities exist is a costly, difficult, and well-nigh impossible task. It follows that, in this latter case, the sum of 1s. 2d. per head is not adequate to supply a good dinner, including tables, chairs, tablecloths, knives and forks, and all other fittings essential to success and comfort. We fear, from what we hear, that this fact has not been grasped at the moment by the central committee at the Mansion House, and in consequence great dissatisfaction may result to the poor, with attendant unpopularity to the dinner and discredit to those who are responsible for the arrangements.

Queen Alexandra's dinner to the poor at the time of the Diamond Jubilee has taught several lessons. In the better-managed parishes and districts where the Queen's dinners were given on that occasion, the clergy and local authorities had the wisdom to spend some money in plant, and they so secured, in connection with their schools and halls, tables, linen, knives and forks, and other fittings, so that they are in a position to provide a good dinner or other meal at the smallest cost as occasion may require. We hope that, wherever this course has not yet been followed, it may be established in connection with the King's dinner through the liberality of those residents in each district who wish to co-operate in carrying out the King's wish in the happiest possible manner. The prosperous can usefully lend a hand by providing the requisites of the table, and presenting them to the clergy or local authorities who manage the buildings in which the King's dinner will be held.

Another difficulty is created from the feeling which has got abroad that the respectable poor will have to sit down with the riff-raff, and so may be annoyed or suffer great discomfort. Here again we have an illustration of the relative efficiency or otherwise of the administration of the affairs of the various parishes and districts throughout London where the dinners are to be held. In those which are best administered arrangements have been made to group the poor, so that the respectable poor will dine together at one place, those in receipt of parish relief at another place, and the rougher classes at a third place. In this way every difficulty of the kind has been successfuly overcome. But, where no such organisation has as yet been thought of or attempted, trouble must ensue. Again, one borough council have made an offer to the clergy to give them a certain number of invitations to the King's dinner to distribute amongst their poor. In one parish of 2,000 inhabitants 60 invitations have been offered, with the result that the vicar has decided that none but the very aged, infirm, and bedridden can be invited to the King's feast, and the great majority of the poor will have no share or part in it. Yet the same borough council have invited everybody in the district who wishes to be present at the dinner on July 5th to send in their names to the Vestry Clerk, showing that the arrangements have not been thought out with sufficient care, and that dissatisfaction and misapprehension must result. It does not augur well for the success of the King's dinner, nor is it likely to promote public appreciation of the ability of the borough councils, who will be held responsible for failure, that some of their present arrangements should be calculated to cause an amount of ill-feeling and discontent, accompanied by bitter disappointment to the majority of the poor, and the fostering of grumbling and jealousy.

The end of the matter appears to us to be this. It is essential that Sir Thomas Lipton, who has been put prominently forward as the head of the organisation of this dinner, should make it his business to visit every district of the metropolis and personally inspect the arrangements which are being made, so that they may be supplemented and improved to the requisite extent. If this is not done we have good reason for fearing that the King's dinner may produce an amount of unpopularity and dissatisfaction which is likely to rankle and continue for months, and so may cause serious mischief. Of course it is a hugely difficult task to handle so vast a population of poor people as are to be found in London to-day. But those who have undertaken the task must be held responsible for its proper fulfilment, and it is the duty of the Press to call attention in time to the dangers which we have indicated, for otherwise the kindly thought of the King may fail in its fulfilment, not from any fault of His Majesty, but from a want of administrative ability on the part of those who have been charged with the duty of making arrangements for the feast.

